# Outcomes following large joint arthroplasty: does socio-economic status matter?

**DOI:** 10.1186/1471-2474-15-148

**Published:** 2014-05-06

**Authors:** Michelle M Dowsey, Mandana Nikpour, Peter FM Choong

**Affiliations:** 1 Department of Orthopaedics and The University of Melbourne Department of Surgery, St. Vincent’s Hospital Melbourne, 41 Victoria Parade Fitzroy, Victoria 3065, Australia; 2 The University of Melbourne Departments of Medicine and Rheumatology, St. Vincent’s Hospital Melbourne, 41 Victoria Parade Fitzroy, Victoria 3065, Australia

**Keywords:** Knee, Hip, Arthroplasty, Socioeconomic status, Outcome

## Abstract

**Background:**

We sought to determine whether socio-economic status (SES) is an independent predictor of outcome following total knee (TKR) and hip (THR) replacement in Australians.

**Methods:**

In this prospective cohort study, we included patients undergoing TKR and THR in a public hospital in whom baseline and 12-month follow-up data were available. SES was determined using the Australian Bureau of Statistics ‘Index of Relative Advantage and Disadvantage’. Other independent variables included patients’ demographics, comorbidities and procedure-related variables. Outcome measures were the International Knee Society Score and Harris Hip Score pain and function subscales, and the Short Form Health Survey (SF-12) physical and mental component scores.

**Results:**

Among 1,016 patients undergoing TKR and 835 patients undergoing THR, in multiple regression analysis, SES score was not independently associated with pain and functional outcomes. Female sex, older age, being a non-English speaker, higher body mass index and presence of comorbidities were associated with greater post-operative pain and poorer functional outcomes following arthroplasty. Better baseline function, physical and mental health, and lower baseline level of pain were associated with better outcomes at 12 months. In univariate analysis, for TKR, the improvement in SF-12 mental health score post arthroplasty was greater in patients of lower SES (3.8 ± 12.9 versus 1.5 ± 12.2, p = 0.008), with a statistically significant inverse association between SES score and post-operative SF-12 mental health score in linear regression analysis (coefficient−0.28, 95% CI: −0.52 to −0.04, p = 0.02).

**Conclusions:**

When adjustments are made for other covariates, SES is not an independent predictor of pain and functional outcome following large joint arthroplasty in Australian patients. However, relative to baseline, patients in lower socioeconomic groups are likely to have greater mental health benefits with TKR than more privileged patients. Large joint arthroplasty should be made accessible to patients of all SES.

## Background

Joint replacement surgery is one of the most common and costly surgical procedures performed in developed countries [1-3]. Despite technical advances in orthopaedic surgery, there remain many patient-related factors that influence the outcome of large joint arthroplasty [4-8]. Previous studies have indicated that lower socioeconomic status (SES) may be associated with worse outcomes post total knee (TKR) and hip (THR) replacement [9-14]. Possible reasons for this include low motivation, poor health literacy, nutrition, housing and living conditions among those in lower socio-economic groups. The impact of SES on the outcomes of arthroplasty has important implications in relation to selection of suitable patients for joint replacement, and strategies such as psychosocial interventions to optimize the outcomes of this procedure.

Due to differences in socio-economic fabric, ethnic composition, health care systems and cultural expectations, the relative importance of SES as a predictor of outcome post TKR and THR may differ among nations. The Australian ‘public’ health care system provides government subsidized health care for all Australians. Hospital-based services, including elective surgical procedures such as arthroplasty, are free of charge to patients. Accordingly, Australians of all socioeconomic backgrounds access these services. In this study, we sought to determine the association between SES and outcomes in Australian patients undergoing TKR or THR in a specialized ‘public hospital’ care setting.

## Methods

### Setting and patients

This study was conducted at St. Vincent’s Hospital, a 460-bed university-affiliated tertiary referral centre situated in the central metropolitan region of Melbourne, Australia. All patients who underwent primary TKR or THR (arthroplasty), between 1 January 2006 and 31 December 2009, in whom baseline and 12-month follow-up data were available, were eligible for enrolment into the study. For patients who underwent staged bilateral joint replacement, only the second procedure was included in the analyses. The study was approved by the Human Research Ethics Committee of St. Vincent’s Hospital Melbourne.

### Data collection

Patients attended a multidisciplinary pre-admission clinic within eight weeks prior to surgery, wherein ‘baseline’ pre-operative data were collected according to a standardized protocol. Data included demographics, diagnosis and co-morbidities. Patients were then followed through their procedure and details such as prosthesis type and peri-operative interventions were recorded. Health related questionnaires were administered to patients pre- and 12 months post-surgery. These patient-reported measures were the International Knee Society Score (IKSS) [15], the Harris Hip Score (HHS) [16] and the Short Form Health Survey (SF-12) [17]. Follow-up captured outcomes including death, re-hospitalization and complications.

### Main independent variable

The main independent (‘predictor’) variable in this study was socio-economic status (SES). In order to determine SES, the residential postcode of each patient was matched to the corresponding Australian Bureau of Statistics (ABS) ‘Index of Relative Advantage and Disadvantage’ , which incorporates variables such as income, education, occupation, housing and employment [18]. This index was developed using data from the entire Australian population surveyed in the most recent nation-wide ‘census’. This index summarizes the socio-economic characteristics of subjects within an area [18], and is reported as a ranked score from one to ten (ten equal deciles), with one representing the most disadvantaged and ten the most advantaged areas.

### Covariates

#### Patient characteristics

All analyses were adjusted for age, sex, body mass index (BMI), aetiology of knee or hip joint disease, non-English speaking background, history of contralateral joint replacement and presence of comorbidities. Comorbidities were measured using the Charlson co-morbidity Index (CCI) [19]. The CCI is a widely used and validated measure consisting of a weighted scale of 17 co-morbidities expressed as a summative score [19]. The CCI was calculated using co-morbidity data recorded during the pre-operative medical assessment and the anaesthetic assessment on the day of surgery. The CCI was subsequently adjusted for age [20].

#### Procedure-related variables

For TKRs, analyses were adjusted for prosthesis type (cruciate retaining, posterior stabilizing or ultra-congruent) and patellar resurfacing. For THRs, analyses were adjusted for cemented versus non-cemented prosthesis. Post-operative complications were also recorded.

### Outcome variables

For TKR’s, the outcome variables were IKSS pain (IKSS_Pain_) and function (IKSS_Function_) subscales, and SF-12 recorded at 12 months post arthroplasty. The IKSS is a validated scoring system for TKR [21], and good inter-observer reproducibility for the pain and function subscales has been demonstrated [22]. IKSS_Pain_ is assessed on a subscale that ranges from no pain (50 points) to mild/occasional (45 points), mild on stairs (40 points), mild on walking and stairs (30 points), moderate occasional (20 points), moderate continuous (10 points) and severe (0 points) pain. The IKSS_Function_ is based on walking distance, ability to climb stairs and the use of gait aids; the score ranges from 0–100, with a lower score indicating greater functional limitation.

The SF-12 is a multipurpose, generic measure of health status that measures eight health concepts from which two distinct component scores are derived; the physical component score (PCS) and the mental component score (MCS) [17]. Both component scores are designed to have a mean of 50 and a standard deviation of 10, with lower scores indicating greater physical or mental health impairment [23]. A score of ≥ 50 indicates no impairment; 40–49 mild impairment; 30–39 moderate impairment; and < 30 severe impairment. The SF-12 is commonly used to measure physical and mental wellbeing in the clinical setting [24] and specifically in both TKR and THR [25,26]; it is validated for use in the Australian population [27].

For THRs, the outcome variables were HHS pain (HHS_Pain_), and function (HHS_Function_) subscales and SF-12 at 12 months post arthroplasty. HHS_Pain_ and HHS_Function_ are two of the four subscales of the HHS [16]. HHS_Pain_ score ranges from no pain (44 points) to slight (40 points), mild (30 points), moderate (20 points), severe (10 points) pain, and disabled (0 points). The functional score range is from 0–47 and assessment is based on walking distance, ability to climb stair, the use of gait aids, limping, ability to don shoes and socks, catch public transport and sit. A lower score indicates greater functional limitation.

All patients were mailed health questionnaires to complete and return at their pre-surgery or follow-up assessments. Patients who did not return during their assessment were contacted by telephone by a person independent of the research team.

### Statistical analysis

For each of TKR’s and THR’s, patient characteristics and procedure-related variables were summarized as mean and standard deviation (mean ± SD) for continuous variables, and proportions (percentages) for categorical variables. For each of the TKR and THR datasets, patients were divided into those with ‘low’ SES score ≤ 5 (SES_Low_) and ‘high’ SES score ≥ 6 (SES_High_), and univariate methods (t-test, chi-square and analysis of variance) were used to compare characteristics in the two groups.

Linear regression models were run to determine the independent predictors (including SES score on a continuous scale from 1 to 10) of each of the outcome variables: IKSS_Pain_, IKSS_Function_, PCS_Knee_ and MCS_Knee_, HHS_Pain_, HHS_Function_, PCS_Hip_ and MCS_Hip_. For each outcome, the dependent variable was the outcome measure at 12 months post-surgery, with the pre-operative ‘baseline’ measures included as independent variables. Linearity was tested using plots of observed versus predicted values and plots of residuals versus predicted values in order to ensure that the linearity assumption was satisfied for regression modelling. We also ran models wherein the dependent variable was the *change* in outcome measure (post-operative minus pre-operative); the results were very similar to the first set of models and therefore we have not presented these here (see Additional file 1).

Results of linear regression were presented as a coefficient (parameter estimate) for each independent variable, with corresponding 95% confidence interval (95% CI) and p value. P values ≤ 0.05 were deemed statistically significant. All analyses were performed using STATA 11 software (StataCorp LP, Texas, USA).

## Results

A total of 1,212 TKR’s in 1,065 patients and 982 THR’s in 891 patients were performed during the study period. For patients who underwent staged bilateral joint replacement the second procedure was included in the analysis. A total of 105 patients were excluded due to; deceased prior to 12 months follow-up (12 knees, 12 hips), underwent simultaneous procedure (1 knee), revision of prosthesis prior to 12 months follow-up (3 knees, 9 hips), underwent contralateral or other large joint arthroplasty within 12 months (15 knees, 20 hips), did not complete surveys at both time-points (18 knees, 15 hips). Therefore follow-up data was available for 1016/1065 (95.4%) of patients who underwent TKR and 835/891 (93.7%) of patients who underwent THJR.

### Knee arthroplasty analyses

**Figure 1:**
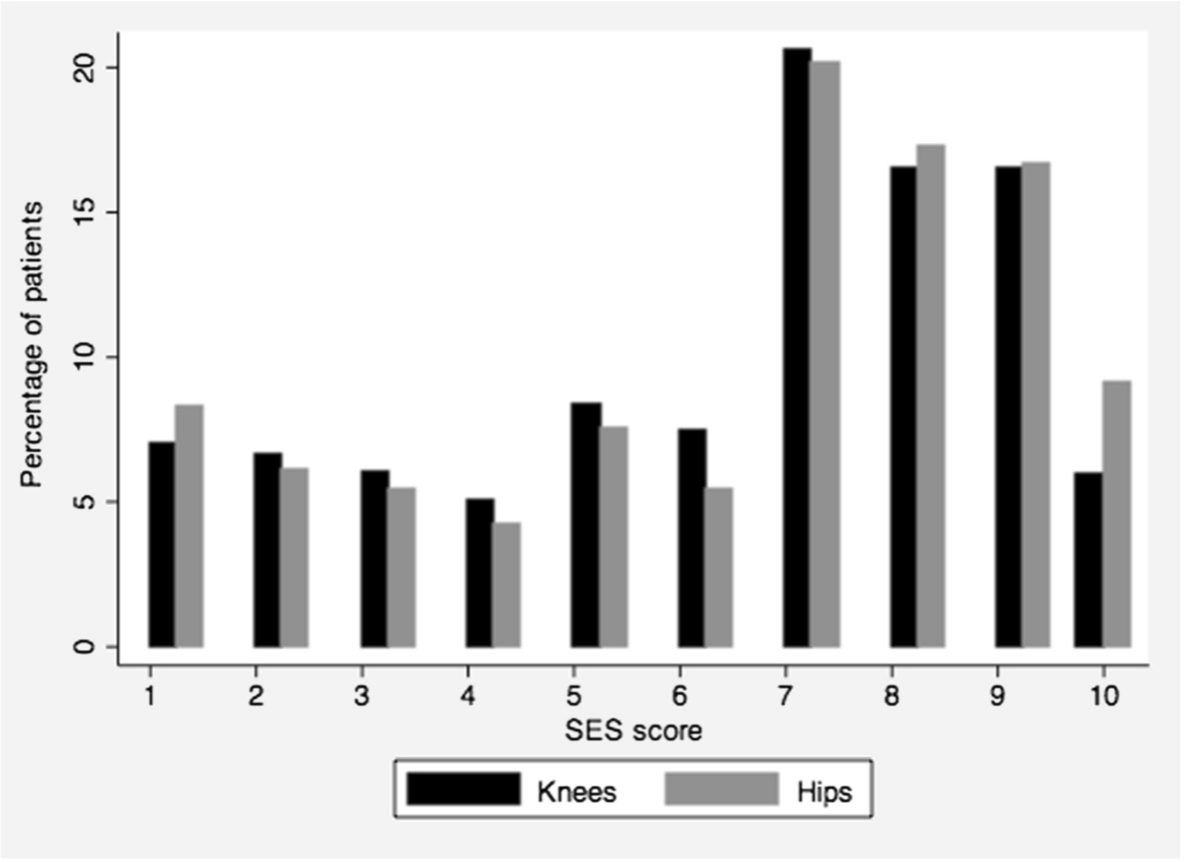
**Distribution of SES scores.** Distribution of SES scores among patients (with a higher score representing socioeconomic advantage).

Characteristics of the 1,016 patients who underwent TKR are summarized in Tables 1 and 2. The distribution of SES scores among patients is presented in Figure 1. The mean ± SD SES score was 6.3 ± 2.6, with a median of 7.

**Table 1:** Patient characteristics (categorical variables)

	**Knee replacements n (%)**	**Hip replacements n (%)**
Total number of patients	1016 (100%)	835 (100%)
Sex:		
Female	688 (67.7%)	502 (60.1%)
Male	328 (32.3%)	333 (39.9%)
Obesity^¶^	641 (63.1%)	371 (44.4%)
Aetiology:		
OA	957 (94.2%)	726 (87.0%)
RA	55 (5.4%)	33 (4.0%)
AVN	4 (0.4%)	50 (6.0%)
CHD	-	26 (3.1%)
Contralateral joint replacement	325 (32.0%)	204 (24.4%)
Prosthesis type:^§^		-
Cruciate retaining	400 (39.4%)	
Posterior stabilising	580 (57.1%)	
Ultra congruent	36 (3.5%)	
Patella resurfaced^§^	315 (31.0%)	-
Cementation:^∞^	-	
Uncemented		148 (17.7%)
Hybrid		617 (73.9%)
Totally cemented		70 (8.4%)
Diabetes	196 (19.3%)	104 (12.5%)
Hypertension	639 (62.9%)	440 (52.7%)
AMI/IHD/CCF	116 (11.4%)	89 (10.7%)
Asthma/COAD	167 (16.4%)	119 (14.3%)
Cancer	69 (6.8%)	58 (7.0%)
Smoker:		
Ex-	235 (23.1%)	236 (28.3%)
Current	60 (5.9%)	91 (10.9%)
Complication	222 (21.9%)	222 (26.6%)
Non-English speaking^□^	154 (15.2%)	84 (10.1%)
Pre-op physical impairment:**		
None	6 (0.6%)	2 (0.2%)
Mild	45 (4.4%)	20 (2.4%)
Moderate	208 (20.5%)	140 (16.8%)
Severe	757 (74.5%)	673 (80.6%)
Post-op physical impairment:**		
None	170 (16.7%)	192 (23.0%)
Mild	248 (24.4%)	219 (26.2%)
Moderate	306 (30.1%)	237 (28.4%)
Severe	292 (28.7%)	187 (22.4%)
Pre-op mental distress:***		
None	525 (51.7%)	359 (43.0%)
Mild	203 (20.0%)	197 (23.6%)
Moderate	228 (22.4%)	201 (24.1%)
Severe	60 (5.9%)	78 (9.3%)
Post-op mental distress:***		
None	593 (58.4%)	516 (61.8%)
Mild	222 (21.9%)	168 (20.1%)
Moderate	162 (15.9%)	109 (13.1%)
Severe	39 (3.8%)	42 (5.0%)

*Abbreviations*: *OA* Osteoarthritis, *RA* Rheumatoid Arthritis, *AVN* Avascular necrosis, *CHD* Congenital hip dysplasia, *AMI* acute myocardial infarction, *IHD* ischemic heart disease, *CCF* congestive cardiac failure, *COAD* chronic obstructive airways disease, *pre-op* pre-operative, *post-op* post-operative.

^¶^Obesity defined as body mass index (BMI) ≥ 30.

^§^Applies only to knee replacements.

^∞^Applies only to hip replacements.

^□^Need interpreter in order to communicate with an English speaker.

**Based on categories of SF12_physical_; score of ≥ 50 indicates no impairment; 40–49 mild impairment; 30–39 moderate impairment; and < 30 severe impairment.

***Based on categories of SF12_mental_; score of ≥ 50 indicates no distress; 40–49 mild distress; 30–39 moderate distress; and < 30 severe distress.

**Table 2:** Patient characteristics (continuous variables)

**Characteristic**	**Knee replacements (n = 1016)**	**Hip replacements (n = 835)**
	**Mean ± SD**	**Mean ± SD**
Age (years)	70.3 ± 8.6	68.4 ± 9.9
BMI (kg/m^2^)^¶^	32.4 ± 5.9	29.8 ± 5.7
SES score^□^	6.3 ± 2.6	6.4 ± 2.7
Age-adjusted CCI*	1.8 ± 2.2	1.5 ± 2.1
Pre-op IKSS_Pain_^∞^	5.4 ± 8.2	-
Post-op IKSS_Pain_^∞^	35.2 ± 15.3	-
Change in IKSS_Pain_^∞^	29.8 ± 16.4	-
Pre-op IKSS_Function_^**^	36.2 ± 19.5	-
Post-op IKSS_Function_^**^	58.5 ± 25.8	-
Change in IKSS_Function_^**^	22.3 ± 24.3	-
Pre-op PCS^§^	27.1 ± 6.3	26.2 ± 5.5
Post-op PCS^§^	37.8 ± 10.7	40.0 ± 11.1
Change in PCS^§^	10.6 ± 10.9	13.8 ± 11.4
Pre-op MCS^¶^	48.7 ± 11.8	46.6 ± 12.0
Post-op MCS^¶^	50.9 ± 10.9	51.2 ± 10.8
Change in MCS^¶^	2.3 ± 12.5	4.6 ± 12.9
Pre-op HHS_Pain_^#^	-	12.0 ± 4.6
Post-op HHS_Pain_^#^	-	38.6 ± 9.0
Change in HHS_Pain_^#^	-	45.1 ± 17.5
Pre-op HHS_Function_^¢^	-	17.1 ± 9.1
Post-op HHS_Function_^¢^	-	34.2 ± 10.7
Change HHS_Function_^¢^	-	17.1 ± 10.9

*Abbreviations*: *BMI* Body Masss Index, *SES score* Socioeconomic status score, *CCI* Charlson Comorbidity Index, *pre-op* pre-operative, *post-op* post-operative, *IKSS*_*Pain*_ International Knee Society Pain Score, *IKSS*_*Function*_ International Knee Society Function Score, *PCS* Short Form 12 Physical Component Score, *MCS* Short Form 12 Mental Component Score, *HHS*_*Pain*_ Harris Hip Pain Score, *HHS*_*Function*_ Harris Hip Function Score.

^¶^BMI - Body Mass Index (weight [Kg]/height [m]^2^).

^□^SES score – Socioeconomic status score, (0 to 10) with a higher score representing socioeconomic advantage.

*CCI – Charlson Comorbidity Index (0–43, age adjusted), with a higher score indicating a greater comorbidity burden.

^∞^IKSS_Pain_ – International Knee Society Pain Score (0 to 50) with a higher score representing less pain.

^**^IKSS_Function_ – International Knee Society Function Score (0 to 100) with a higher score representing better function.

^§^PCS - score of ≥ 50 indicates no impairment; 40–49 mild impairment; 30–39 moderate impairment; and < 30 severe impairment.

^¶^MCS - score of ≥ 50 indicates no distress; 40–49 mild distress; 30–39 moderate distress; and < 30 severe distress.

^#^HHS_Pain_ – Harris Hip Pain Score (0 to 44) with a higher score representing less pain.

^¢^HHS_Function_ – Harris Hip Function Score (0 to 47) with a higher score representing better function.

The Mean ± SD CCI at surgery was 1.8 ± 2.2. The mean ± SD number of comorbidities per patient was 2.7 ± 1.5. Complications occurred in 222 (21.9%) and included medical (n = 106), wound (n = 57) and orthopaedic (n = 40) complications; 19 patients had a minor complication that did not require significant intervention or substantially prolong length of stay. An unplanned readmission was required in 88 (8.7%) patients and an additional unplanned procedure was required in 56 (5.5%) patients; the most common indications for this were wound complications.

#### Association of SES and knee arthroplasty

SES scores were initially categorized into ‘low’ (SES_Low_; SES score 5 or less) and ‘high’ (SES_High_; SES score 6 or higher) for descriptive purposes and for univariate analysis. In univariate analysis (Table 3), a significantly higher proportion of patients in the SES_Low_ category were obese (69.0% vs 60.2%, p = 0.02) than in the SES_High_ category. Whilst patients in the SES_Low_ category had a higher level of pre-operative physical function measured using SF12_Physical_ (PCS_Knee_; 27.9 ± 6.8 vs 26.8 ± 6.0, p = 0.008), they had a lower level of pre-operative mental health measured using SF12_Mental_ (MCS_Knee_; 47.2 ± 12.0 vs 49.4 ± 11.6, p = 0.005) than the SES_High_ category. Overall, the improvement in mental health (MCS_Knee_) post surgery was significantly greater for the SES_Low_ (3.8 ± 12.9 vs 1.5 ± 12.2, p = 0.008) than the SES_High_ category.

**Table 3:** **Patient characteristics according to ‘low’ SES score ≤ 5 (SES**_**Low**_**) **_**or **_**‘high’ SES score ≥ 6 (SES**_**High**_**)**

	**Knee replacements (n = 1,016)**			**Hip replacements (n = 835)**		
	**SES**_**Low **_**(n = 335)**	**SES**_**High **_**(n = 681)**	***p***	**SES**_**Low **_**(n = 263)**	**SES**_**High **_**(n = 572)**	***p***
	**Mean ± SD or n (%)**	**Mean ± SD or n (%)**		**Mean ± SD or n (%)**	**Mean ± SD or n (%)**	
Sex:						
Female	236 (70.5%)	452 (66.4%)	0.19	147 (55.9%)	355 (62.1%)	0.09
Male	99 (29.6%)	229 (33.6%)		116 (44.1%)	217 (37.9%)	
Age (years)	69.1 ± 8.6	70.9 ± 8.5	0.002	67.8 ± 10.2	68.7 ± 9.8	0.19
BMI (kg/m^2^)^¶^	33.0 ± 5.7	32.1 ± 6.0	0.02	30.2 ± 5.1	29.7 ± 5.9	0.44
Obesity^¥^	231 (69.0%)	410 (60.2%)	0.007	126 (47.9%)	245 (42.8%)	0.17
Aetiology:						0.09
OA	312 (93.1%)	645 (94.7%)	0.5	222 (84.4%)	504 (88.1%)	
RA	22 (6.6%)	33 (4.9%)		17 (6.5%)	16 (2.8%)	
AVN	1 (0.3%)	3 (0.4)		16 (6.1%)	34 (5.9%)	
CHD	-	-		8 (3.0%)	18 (3.2%)	
Contralateral joint replacement^□^	102 (30.5%)	223 (32.8%)	0.46	64 (24.3%)	140 (24.5%)	0.97
Prosthesis type:			0.12	-	-	-
Cruciate retaining	123 (36.7%)	277 (40.7%)				
Posterior stabilizing	195 (58.2%)	385 (56.5%)				
Ultra congruent	17 (5.1%)	19 (2.8%)				
Patella resurfaced	108 (32.2%)	207 (30.4%)	0.55	-	-	-
Cementation:						0.82
Uncemented	-	-	-	48 (18.3%)	100 (17.5%)	
Hybrid				191 (72.6%)	426 (74.5%)	
Totally cemented				24 (.1%)	46 (8.0%)	
Age-adjusted CCI*	1.7 ± 2.1	1.8 ± 2.2	0.55	1.6 ± 2.1	1.5 ± 2.1	0.70
Complication	64 (19.1%)	158 (23.2%)	0.14	67 (25.5%)	155 (27.1%)	0.62
Non-English speaking	52 (15.5%)	102 (15.0%)	0.82	29 (11.0%)	55 (9.7%)	0.54
Pre-op IKSS_Pain_^∞^	5.7 ± 8.5	5.2 ± 8.1	0.35	-	-	-
Post-op IKSS_Pain_^∞^	35.0 ± 15.3	35.3 ± 15.3		-	-	-
Change in IKSS_Pain_^∞^	29.2 ± 16.9	30.1 ± 16.1	0.44	-	-	-
Pre-op IKSS_Function_^**^	36.7 ± 19.5	35.9 ± 19.6	0.57	-	-	-
Post-op IKSS_Function_^**^	58.0 ± 24.9	58.7 ± 26.3	0.65	-	-	-
Change in IKSS_Function_^**^	21.3 ± 23.1	22.8 ± 24.8	0.35	-	-	-
Pre-op PCS^§^	27.9 ± 6.8	26.8 ± 6.0	0.008	26.9 ± 6.1	25.9 ± 5.2	0.012
Post-op PCS^§^	37.8 ± 11.0	37.7 ± 10.6	0.88	39.7 ± 11.2	40.1 ± 11.0	0.58
Change in PCS^§^	10.0 ± 11.3	10.0 ± 10.6	0.17	12.8 ± 11.7	14.3 ± 11.3	0.08
Pre-op MCS^¶^	47.2 ± 12.0	49.4 ± 11.6	0.005	45.6 ± 12.5	47.0 ± 11.8	0.11
Post-op MCS^¶^	50.9 ± 11.0	50.9 ± 10.9	0.99	51.5 ± 10.7	51.0 ± 10.8	0.54
Change in MCS^¶^	3.8 ± 12.9	1.5 ± 12.2	0.008	5.9 ± 13.3	4.0 ± 12.7	0.047
Pre-op HHS_Pain_^#^	-	-	-	11.9 ± 4.6	12.1 ± 4.6	0.72
Post-op HHS_Pain_^#^	-	-	-	38.2 ± 9.6	38.8 ± 8.7	0.34
Change in HHS_Pain_^#^	-	-	-	26.2 ± 10.3	26.7 ± 9.5	0.47
Pre-op HHS_Function_^¢^	-	-	-	17.1 ± 9.7	17.0 ± 8.8	0.93
Post-op HHS_Function_^¢^	-	-	-	34.3 ± 10.3	34.2 ± 10.9	0.89
Change in HHS_Function_^¢^	-	-	-	17.2 ± 11.3	17.1 ± 10.8	0.94

*Abbreviations*: *BMI* Body Mass Index, *OA* Osteoarthritis, *RA* Rheumatoid Arthritis, *AVN* Avascular necrosis, *CHD* Congenital hip dysplasia, *SES score* Socioeconomic status score, *CCI* Charlson Comorbidity Index, *IKSS*_*Pain*_ International Knee Society Pain Score, *pre-op* pre-operative, *post-op* post-operative, *IKSS*_*Function*_ International Knee Society Function Score, *SF12*_*Physical*_ Short Form 12 physical component, *SF12*_*mental*_ Short Form 12 mental component, *HHS*_*Pain*_ Harris Hip Pain Score, *HHS*_*Function*_ Harris Hip Function Score.

^¶^BMI - Body Mass Index (weight [Kg]/height [m]^2^).

^¥^Obesity defined as BMI ≥ 30.

^□^SES score – Socioeconomic status score, (0 to 10) with a higher score representing socioeconomic advantage.

*CCI – Charlson Comorbidity Index (0–43, age adjusted), with a higher score indicating a greater comorbidity burden.

^∞^IKSS_Pain_ – Knee Society Pain Score (0 to 50) with a higher score representing less pain.

^**^IKSS_Function_ – Knee Society Function Score (0 to 100) with a higher score representing better function.

^§^PCS - score of ≥ 50 indicates no impairment; 40–49 mild impairment; 30–39 moderate impairment; and < 30 severe impairment.

^¶^MCS - score of ≥ 50 indicates no distress; 40–49 mild distress; 30–39 moderate distress; and < 30 severe distress.

^#^HHS_Pain_ – Harris Hip Pain Score (0 to 44) with a higher score representing less pain.

^¢^HHS_Function_ – Harris Hip Function Score (0 to 47) with a higher score representing better function.

In linear regression modelling (Tables 4 and 5), SES score was not independently associated with post-operative IKSS_Pain_, IKSS_Function_ or PCS_Knee._ However, there was a statistically significant inverse association between SES score and post-operative MCS_Knee_ (coefficient −0.28, 95% CI: −0.52 to −0.04, p = 0.02), indicating that after adjustment for baseline MCS_Knee_, patients in lower socioeconomic groups tended to have better mental health scores post arthroplasty.

**Table 4:** **Predictors of IKSS**_**Pain**_**, IKSS**_**Function, **_**HHS**_**Pain **_**and HHS**_**Function **_**(multiple linear regression models)**

	**IKSS**_**Pain**_	**IKSS**_**Function**_	**HHS**_**Pain**_	**HHS**_**Function**_
**Variables**	**Coefficient (95% CI) *p***	**Coefficient (95% CI) *p***	**Coefficient (95% CI) *p***	**Coefficient (95% CI) *p***
Female sex	−1.85 (−3.91:0.22) 0.08	**−9.16** (−12.17:-6.14) < 0.0001	0.72 (−0.53:1.97) 0.26	−1.27 (−2.59:0.05) 0.06
Age (years)	0.07 (−0.04:0.19) 0.22	**−0.38** (−0.55:-0.21) < 0.0001	**0.09** (0.01:0.17) 0.04	**−0.20** (−0.29:-0.12) < 0.0001
SES score^□^	0.08 (−0.27:0.44) 0.64	0.17 (−0.35:0.68) 0.52	0.07 (−0.15:0.30) 0.51	0.06 (−0.18:0.29) 0.63
BMI^¶^	0.07 (−0.09:0.23) 0.40	**−0.48** (−0.71:-0.24) < 0.0001	0.04 (−0.07:0.15) 0.45	**−0.23** (−0.35:-0.12) < 0.0001
Prosthesis type^§^	−1.58 (−3.24:0.09) 0.06	**−4.05** (−6.47:-1.62) 0.001	-	-
Cemented prosthesis^$^	-	-	−0.46 (−1.95:1.04) 0.55	**2.35** (0.77:3.93) 0.004
Complication	**−4.35** (−6.58:-2.12) < 0.0001	**−5.26** (−8.51:-2.02) 0.002	−0.43 (−1.83:0.97) 0.54	−0.68 (−2.16:0.80) 0.37
Non-English speaking	**−4.83** (−7.46:-2.21) < 0.0001	**−7.34** (−11.17:-3.52) < 0.0001	−0.67 (−2.69:1.34) 0.51	−1.09 (−3.22:1.04) 0.32
Age-adjusted CCI*	−**0.72** (−1.17:-0.28) 0.001	**−1.25** (−1.90:-0.60) < 0.0001	−**0.34** (−0.65:-0.03) 0.03	**−0.85** (−1.18:0.53) < 0.0001
Pre-op IKSS_Pain_^∞^	**0.16** (0.03:0.28) 0.012	**−0.19** (−0.37:-0.02) 0.032	-	-
Pre-op IKSS_Function_^**^	−0.03 (−0.08:0.03) 0.31	**0.38** (0.30:0.46) < 0.0001	-	-
Pre-op HHS_Pain_^#^	-	-	0.02 (−0.12:0.16) 0.79	**−0.16** (−0.31:0.01) 0.04
Pre-op HHS_Function_^¢^	-	-	0.01 (−0.07:0.09) 0.81	**0.34** (0.26:0.42) < 0.0001
Pre-op PCS^§^	**0.24** (0.07:0.40) 0.005	**0.53** (0.29:0.77) < 0.0001	**0.23** (0.11:0.35) < 0.000	**0.20** (0.06:0.33) 0.003
Pre-op MCS^¶^	**0.18** (0.10:0.27) < 0.0001	**0.25** (0.13:0.38) < 0.0001	**0.13** (0.07:0.19) < 0.0001	**0.10** (0.04:0.16) 0.001

*Abbreviations*: *SES score* Socioeconomic status score, *BMI* Body Mass Index, *CCI* Charlson Comorbidity Index, *pre-op* pre-operative, *post-op* post-operative, *IKSS*_*Pain*_ International Knee Society Pain Score, *IKSS*_*Function*_ International Knee Society Function Score, *HHS*_*Pain*_ Harris Hip Pain Score, *HHS*_*Function*_ Harris Hip Function Score, *SF12*_*Physical*_ Short Form 12 physical component, *SF12*_*mental*_ Short Form 12 mental component.

^□^SES score – Socioeconomic status score, (0 to 10) with a higher score representing socioeconomic advantage.

^¶^BMI - Body Mass Index (weight [Kg]/height [m]^2^).

^§^Coefficient relates to posterior stabilizing or ultra-congruent procedure (compared with cruciate retaining).

^$^Coefficient relates to cemented (hybrid or totally cemented) compared with uncemented hip replacement.

*CCI – Charlson Comorbidity Index (0–43, age adjusted), with a higher score indicating a greater comorbidity burden.

^∞^IKSS_Pain_ – Knee Society Pain Score (0 to 50) with a higher score representing less pain.

^**^IKSS_Function_ – Knee Society Function Score (0 to 100) with a higher score representing better function.

^#^HHS_Pain_ – Harris Hip Pain Score (0 to 44) with a higher score representing less pain.

^¢^HHS_Function_ – Harris Hip Function Score (0 to 47) with a higher score representing better function.

^§^PCS - score of ≥ 50 indicates no impairment; 40–49 mild impairment; 30–39 moderate impairment; and < 30 severe impairment.

^¶^MCS - score of ≥ 50 indicates no distress; 40–49 mild distress; 30–39 moderate distress; and < 30 severe distress.

Bold numbers denotes statistical significance (p < 0.05).

**Table 5:** Predictors of SF12-physical (PCS) and SF12-mental (MCS) for the knee and hip datasets (multiple linear regression models)

	**PCS**_**Knee**_	**MCS**_**Knee**_	**PCS**_**Hip**_	**MCS**_**Hip**_
**Variables**	**Coefficient (95% CI) *p***	**Coefficient (95% CI) *p***	**Coefficient (95% CI) *p***	**Coefficient (95% CI) *p***
Female sex	−1.20 (−2.56:0.15) 0.08	−0.19 (−1.59:1.21) 0.79	0.01 (−1.43:1.45) 0.99	**−1.47** (−2.90:-0.04) 0.04
Age (years)	−0.06 (−0.14:0.02) 0.13	**0.11** (0.03:0.19) 0.005	**−0.12** (−0.21:-0.02) 0.02	0.07 (−0.02:0.17) 0.12
SES score^□^	0.05 (−0.18:0.29) 0.65	**−0.28** (−0.52:-0.04) 0.02	0.13 (−0.12:0.39) 0.30	−0.15 (−0.40:0.10) 0.25
BMI^¶^	−0.06 (−0.17:0.04) 0.24	−0.01 (−0.12:0.09) 0.79	−0.11 (−0.24:0.01) 0.08	0.02 (−0.11, 0.14) 0.81
Prosthesis type^§^	**−1.16** (−2.25:0.07) 0.04	−0.99 (−2.12:0.13) 0.08	-	-
Cemented prosthesis^$^	-	-	0.75 (−0.98:2.46) 0.40	−0.12 (−1.82:1.58) 0.89
Complication	**−2.76** (−4.23:-1.30) < 0.0001	**−1.60** (−3.11:0.10) 0.04	−1.31 (−2.93:0.30) 0.11	−0.39 (−1.99:1.20) 0.63
Non-English speaking	−1.23 (−2.96:0.49) 0.16	−1.60 (−3.37:-0.18) 0.08	−0.77 (−3.10:1.55) 0.51	−0.44 (−2.73:1.86) 0.71
Age-adjusted CCI^*^	−**0.72** (−1.01:-0.42) < 0.0001	**−0.36** (−0.67:-0.06) 0.02	−**0.92** (−1.28:-0.57) < 0.0001	**−0.54** (−0.89:-0.19) 0.002
Pre-op KSS_Pain_^∞^	−0.04 (−0.12:0.04) 0.35	0.03 (−0.05:0.11) 0.43	-	-
Pre-op KSS_Function_^**^	**0.07** (0.03:0.11) < 0.0001	−0.00002 (−0.04:0.04) 1.00	-	-
Pre-op HHS_Pain_^#^	-	-	**−0.17** (−0.33:0.001) 0.048	−0.04 (−0.20:0.13) 0.67
Pre-op HHS_Function_^¢^	-	-	**0.14** (0.04:0.23) 0.004	−0.09 (−0.18:0.004) 0.06
Pre-op PCS^§^	**0.43** (0.32:0.54) < 0.0001	0.07 (−0.04:0.18) 0.20	**0.35** (0.21:0.50) < 0.0001	**0.14** (0.001:0.28) 0.049
Pre-op MCS^¶^	**0.16** (0.11:0.22) < 0.0001	**0.36** (0.31:0.42) < 0.0001	**0.20** (0.13:0.26) < 0.0001	**0.35** (0.28:0.41) < 0.0001

*Abbreviations*: *SES score* Socioeconomic status score, *BMI* Body Mass Index, *CCI* Charlson Comorbidity Index, *pre-op* pre-operative, *post-op* post-operative, *IKSS*_*Pain*_ International Knee Society Pain Score, *IKSS*_*Function*_ International Knee Society Function Score, *HHS*_*Pain*_ Harris Hip Pain Score, *HHS*_*Function*_ Harris Hip Function Score, *SF12*_*Physical*_ Short Form 12 physical component, *SF12*_*mental*_ Short Form 12 mental component.

^□^SES score – Socioeconomic status score, (0 to 10) with a higher score representing socioeconomic advantage.

^¶^BMI - Body Mass Index (weight [Kg]/height [m]^2^).

^§^Coefficient relates to posterior stabilizing or ultra-congruent procedure (compared with cruciate retaining).

^$^Coefficient relates to cemented (hybrid or totally cemented) compared with uncemented hip replacement.

^*^CCI – Charlson Comorbidity Index (0–43, age adjusted), with a higher score indicating a greater comorbidity burden.

^∞^KSS_Pain_ – Knee Society Pain Score (0 to 50) with a higher score representing less pain.

^**^KSS_Function_ – Knee Society Function Score (0 to 100) with a higher score representing better function.

^#^HHS_Pain_ – Harris Hip Pain Score (0 to 44) with a higher score representing less pain.

^¢^HHS_Function_ – Harris Hip Function Score (0 to 47) with a higher score representing better function.

^§^PCS - score of ≥ 50 indicates no impairment; 40–49 mild impairment; 30–39 moderate impairment; and < 30 severe impairment.

^¶^MCS - score of ≥ 50 indicates no distress; 40–49 mild distress; 30–39 moderate distress; and < 30 severe distress.

Bold numbers denotes statistical significance (p < 0.05).

#### Regression models for IKSS_Pain_

Results of linear regression modelling for independent determinants of post-operative IKSS_Pain_ are presented in Table 4. Presence of complications, being a non-English speaker and having a higher burden of comorbidities were associated with lower post-operative IKSS_Pain_ score, i.e., *more* pain at 12 months. Having less pre-operative pain and less pre-operative physical impairment and mental distress were associated with higher post-operative IKSS_Pain_ score, i.e., *less* pain at 12 months.

#### Regression models for IKSS_Function_

Results of linear regression modelling for independent determinants of post-operative IKSS_Function_ are presented in Table 4. Female sex, older age, higher BMI, presence of a complication, being a non-English speaker, having a higher burden of comorbidities and lower pre-operative IKSS_Pain_, were associated with lower IKSS_Function_ score i.e., *worse* function at 12 months. Having better function prior to surgery, a cruciate retaining procedure, and less pre-operative physical impairment and mental distress were associated with higher IKSS_Function_ score i.e., *better* function at 12 months.

#### Regression models for PCS_Knee_

Results of linear regression modelling for independent determinants of post-operative PCS_Knee_ are presented in Table 5. Presence of a complication and a higher burden of comorbidities were associated with *worse* physical health at 12 months post-op. Having a higher pre-operative IKSS_Function_ score, a cruciate retaining procedure, and better pre-operative physical and mental health were associated with *better* physical function at 12 months post arthroplasty.

#### Regression models for MCS_Knee_

Results of linear regression modelling for independent determinants of post-operative MCS_Knee_ are presented in Table 5. Complications and comorbidities were associated with *worse* mental health at 12 months. Older age at time of surgery, lower SES score and better pre-operative mental health were associated with *better* mental health at 12 months post arthroplasty.

### Hip arthroplasty analyses

Characteristics of the 835 patients who underwent THR are summarized in Tables 1 and 2. Overall, these patients were similar in profile to those who had TKR, with several notable exceptions. There was a higher proportion of male patients (333 [39.9%]) and proportionately fewer obese patients (371 [44.4%]). In addition to OA, RA and AVN, 26 (3.1%) had THR for congenital hip dysplasia. Fewer patients had a prior contralateral arthroplasty (204 [24.4%]). Compared with patients undergoing TKR, those having THR had proportionately more post-operative complications 222 (26.6%), though most (118 of 222) of these were minor and did not require intervention or significantly impact length of stay. Overall, the complications were medical (n = 70), wound (n = 34) and orthopaedic (n = 19). The number of unplanned readmissions (46 [5.5%]) and additional unplanned procedures (36 [4.3%])) were both lower for those having THR. However, similar to those undergoing TKR, the most common indication for readmissions/unplanned procedures was wound complications.

#### Association of SES and hip arthroplasty

In univariate analysis (Table 3), there were no significant differences in most of the characteristics of patients in the SES_Low_ compared with the SES_High_ category. However, similar to the knee dataset, patients in the SES_Low_ category had a higher level of pre-operative physical function (PCS_Hip_; 26.9 ± 6.1 vs 25.9 ± 5.2, p = 0.012), and a significantly greater improvement in mental health (MCS_Hip_) post arthroplasty than the SES_High_ category (5.9 ± 13.3 vs 4.0 ± 12.7, p = 0.047).

In linear regression modelling (Tables 4 and 5), SES score was not independently associated with post-operative HHS_Pain_, HHS_Function_, PCS_Hip_ or MCS_Hip_.

#### Regression models for HHS_Pain_

Results of linear regression modelling for independent determinants of post-operative HHS_Pain_ are presented in Table 4. Having a higher burden of comorbidities was associated with *more* pain at 12 months. Being older, having less pre-operative physical impairment and mental distress were associated with *less* pain at 12 months.

#### Regression models for HHS_Function_

Results of linear regression modelling for independent determinants of post-operative HHS_Function_ are presented in Table 4. Older age, higher BMI and a higher number of comorbidities were associated with *worse* function at 12 months. Having less pre-operative physical impairment or mental distress, better pre-operative function, and having a cemented THR were associated with *better* function at 12 months.

#### Regression models for PCS_Hip_

Results of linear regression modelling for independent determinants of post-operative PCS_Hip_ are presented in Table 5. Older age and a higher burden of comorbidities were associated with *worse* physical health at 12 months post arthroplasty. Having better pre-operative function, and physical and mental health were associated with *better* physical function at 12 months post arthroplasty.

#### Regression models for MCS_Hip_

Results of linear regression modelling for independent determinants of post-operative MCS_Hip_ are presented in Table 5. Comorbidities were associated with *worse* mental health at 12 months post arthroplasty. Older age at time of surgery and better pre-operative physical and mental health were associated with *better* mental function at 12 months post arthroplasty.

## Discussion

In an Australian setting, we have shown that SES is not an independent determinant of pain and functional outcomes post joint replacement surgery. We have confirmed several important predictors of pain and functional outcome of arthroplasty including age, BMI, comorbidities, pre-operative pain and function, and pre-operative mental health. We have also shown that relative to baseline, patients in lower socioeconomic groups have the greatest improvement in mental health post arthroplasty.

The lack of a predictive association between SES and several key arthroplasty outcome measures in our study contrasts the findings of most other investigators [10,11,13,14]. A large UK study by Jenkins et.al using a SES classification system similar to the ABS ‘SES score’ , with comparable health questionnaires, showed significant differences in SF-36 physical improvement between the least and most “deprived groups” 18 months post THR [13]. A study based in Scotland by Clement et al. reported similar findings [11]. In a smaller study Allen-Butler et al. [10] conducted a secondary analysis of a prospective randomized study originally comparing 2 different hip stems. They also concluded that individual socioeconomic parameters such as education level, household income, as well as being African American were associated with lower Harris Hip Scores up to 2 years post THR [10]. Finally, a study by Schafer et al. also concluded that socioeconomic variables independently predicted response to THR [14].

Only one study to date has reported that lower SES did not appear to affect the outcome of joint replacement; this was a multicentre study conducted in several countries (USA, UK, AU, Canada) in patients undergoing knee arthroplasty [28]. SES data were derived from a pre-operative questionnaire regarding education, income, working status and living arrangements, to allow for direct comparison between countries. Despite reporting a correlation between lower income and worse pre-operative pain and function, there were no differences in post-operative pain and function at 24 months.

There are several possible explanations for the lack of association between SES and pain/function outcomes following arthroplasty in our study. The first relates to the differences in measures of SES used in the various studies to date. Although elements such as level of education and income are common to most measures of SES, there are possible regional differences in the components of this construct. Therefore, the use of the ABS ‘SES score’ , a composite or ‘global’ measure of SES specifically derived for use in Australia, is a methodological strength of our study. However, within each postal address code, there will likely be individuals who have a lower or higher SES than might be expected. While this is a possible limitation of our study, in the Australian population as a whole, the SES score is a reliable indicator of SES.

Although the median SES score among our patients was 7, over 30% (335 [33.0%] of patients undergoing knee replacement and 263 [31.5%] patients undergoing hip replacements) had a SES score of 5 or under, indicating that our centre serves a varied patient population, ranging from the lowest to highest SES.

Another possible explanation for our findings may be that unlike previous studies, our analyses accounted for multiple variables that are known to influence the outcome of arthroplasty. Previous studies have not reported such comprehensive demographic, surgical and outcome data.

A third possible explanation relates to the delivery of care in our centre. Geared towards the equitable delivery of health care to all, the Australian public health care system aims to optimize outcomes of elective surgery through pre-operative assessment and management of comorbidities, and a multi-disciplinary approach to post-operative care and discharge planning. Our results imply that in a specialized high through-put arthroplasty centre such as ours, which serves a diverse patient population, there is capacity to overcome the potential negative effects of socioeconomic disadvantage. This complements the observation that specialist arthroplasty centres report better patient outcomes overall, compared to non-specialist, low through-put centres [29-31].

One possible limitation of our study is that we did not include data for privately insured patients undergoing arthroplasty in the private sector. However, as there is likely to be less variation in SES in private Australian hospitals, with most patients being from high socioeconomic groups, it may not be possible to effectively assess the impact of SES on arthroplasty outcomes in such a setting.

In this study we assessed the post-operative ‘state’ of our patients relative to their pre-operative ‘state’ , by including baseline pain and function scores as covariates in our regression models. While it has been reported by some that those who have worse pain and functional status pre-surgery may experience greater change in scores compared to those who have better pre-surgery status, the literature is inconsistent and a smaller change in score on a fixed ended scale in those with a better pre-surgery status may also simply reflect a ceiling effect [32,33]. Further it could be argued the actual post-operative status is more reflective of the benefit of surgery. Several of the studies cited above also assessed the post-operative status, relative to baseline [10,28] while others chose to report the change in status [11,13,14]. When we performed the analyses using change in scores as the outcome, our findings remained in contrast to others, in that SES did not predict outcomes post-surgery.

While previous studies have indicated that lower socioeconomic status (SES) may be associated with worse outcomes post total knee (TKR) and hip (THR) replacement, we hypothesise that ‘due to differences in socio-economic fabric, ethnic composition, health care systems and cultural expectations, the relative importance of SES as a predictor of outcome post TKR and THR may differ among nations’. Indeed, ours is the first study to evaluate the association between SES and outcome of arthroplasty in the Australian public health care system. Our findings contrast those of most previous reports by showing that SES is *not* an independent predictor of arthroplasty outcome. Further, we show the novel finding that relative to baseline, patients with lower SES have *greater* mental health benefits post arthroplasty than their more privileged counterparts. Our results imply that in a specialised high through-put arthroplasty centre, which serves a diverse patient population, there is capacity to overcome the potential negative effects of socioeconomic disadvantage. This also complements the observation that specialist arthroplasty centres report better patient outcomes overall, compared to non-specialist, low through-put centres.

The only significant association of SES demonstrated in our study was an inverse correlation between SES score and the post-operative SF12 Mental Component Score among patients undergoing knee arthroplasty (MCS_Knee_). This suggests that compared with more privileged counterparts, those in lower socio-economic groups are even more likely to have higher – and better – post-operative mental health scores, relative to their baseline mental health scores. Arthroplasty has been shown to be a ‘life-changing’ procedure, with a substantial impact on the mental health of patients. In our study, the difference in the mental health gains of patients in low versus high socioeconomic groups may be related to differences in patient expectations and whether these expectations are met.

In this study we have identified several important predictors of outcome following large joint arthroplasty. Among patients undergoing TKR, female gender, higher BMI, limited English proficiency and a greater burden of comorbidities, were associated with worse pain and lower function at 12 months; we and others have identified these variables in prior research [4,6,34]. Pre-operative psychological state in particular appears to be an important determinant of pain and functional outcome in TKR [35]. Similar to TKR, important independent predictors of pain and function in THR were comorbidities, BMI and baseline physical and psychological health. Obesity and psychological distress are common in patients presenting for arthroplasty [5,34-36], and studies are currently under way to evaluate the efficacy and cost effectiveness of mental health programs and obesity interventions in these patient groups [37-39].

In our study, the median post-operative PCS_Knee_ was 37.1, meaning that half of our patients still had moderate to severe functional impairment following the procedure. These results are comparable to the findings of others [25]. Overall, the improvement in physical and mental health with THR, as measured by the SF-12, exceeded that seen with TKR. In addition to inherent differences between the two procedures, here we found that patients undergoing knee arthroplasty were more likely to be female, obese, hypertensive and non-English speaking, and to have previously had surgery on the contralateral side, than those undergoing hip arthroplasty. These findings may in part explain the differences observed in the outcomes of the two procedures.

## Conclusions

In summary, we have shown that in a setting such as ours, underprivileged patients do as well in terms of pain and functional outcomes of arthroplasty, and may even have greater mental health gains than more privileged patients. Our findings further support efforts to ensure equity in access to arthroplasty among patients of all SES.

## Abbreviations

AMI: Acute myocardial infarction; AVN: Avascular necrosis; BMI: Body masss index; CCF: Congestive cardiac failure; CCI: Charlson comorbidity index; CHD: Congenital hip dysplasia; COAD: Chronic obstructive airways disease; HHSPain: Harris hip pain score; HHSFunction: Harris hip function score; IHD: Ischemic heart disease; IKSSPain: International knee society pain score; IKSSFunction: International knee society function score; MCS: Short form 12 mental component score; OA: Osteoarthritis; PCS: Short form 12 physical component score; Pre-op: Pre-operative; Post-op: Post-operative; RA: Rheumatoid arthritis; SES: Socioeconomic status.

## Competing interests

The authors declare that they have no competing interests.

## Authors’ contributions

MMD contributed to study design, data collection, interpretation of findings and preparation of the manuscript. MN contributed to study design, data analysis, interpretation of findings and preparation of the manuscript. PFMC contributed to study design, data collection, interpretation of findings and preparation of the manuscript. All authors read and approved the final manuscript.

## Pre-publication history

The pre-publication history for this paper can be accessed here:

http://www.biomedcentral.com/1471-2474/15/148/prepub

## Supplementary Material

Additional file 1 Predictors of change in Pain, Function and Quality of Life Scores 12 months large joint arthroplasty. Click here for file
