# Supplementary material for: Outcomes following large joint arthroplasty: does socio-economic status matter?
Source: BMC Musculoskelet Disord. 2014 May 6;15:148. doi: 10.1186/1471-2474-15-148 (PMC4107720; doi:10.1186/1471-2474-15-148)
Supplement: Additional file 1 — Predictors of change in Pain, Function and Quality of Life Scores 12 months large joint arthroplasty. [file 1471-2474-15-148-S1.docx]

**Additional file 1**

**Predictors of change in Pain, Function and Quality of Life Scores 12 months large joint arthroplasty**

Manuscript title: Socio-Economic Status as a Predictor of Outcome Following Large Joint Arthroplasty

Authors: Michelle M. Dowsey, Mandana Nikpour, Peter F.M. Choong

## Table a) Predictors of IKSS_Pain_, IKSS_Function,_ HHS_Pain_ and HHS_Function_ (multiple linear regression models) using change in scores as the dependent variable

|  | **IKSS_Pain_** | | **IKSS_Function_** | | **HHS_Pain_** | | **HHS_Function_** | | |
| --- | --- | --- | --- | --- | --- | --- | --- | --- | --- |
| **Variables** | coefficient  (95% CI) | *p* | coefficient  (95% CI) | *p* | coefficient  (95% CI) | *p* | coefficient  (95% CI) | *p* |  |
| Female sex | -1.03  (-3.30, 1.23) | 0.37 | **-5.43**  (-8.73, -2.13) | 0.001 | 1.30  (-0.08, 2.71) | 0.07 | -0.05  (-1.55, 1.44) | 0.95 |  |
| Age | 0.01  (-0.12, 0.14) | 0.91 | -0.10  (-0.29, 0.09) | 0.30 | 0.08  (-0.02, 0.17) | 0.12 | -0.04  (-0.14, -0.009) | 0.48 |  |
| SEIFA^⌘^ | 0.14  (-0.25, 0.52) | 0.49 | 0.19  (-0.38, 0.76) | 0.51 | 0.06  (-0.19, 0.30) | 0.64 | 0.13  (-0.13, 0.39) | 0.33 |  |
| BMI^¶^ | 0.05  (-0.14, 0.23) | 0.63 | **-0.39**  (-0.66, -0.12) | 0.005 | 0.01  (-0.11, 0.13) | 0.87 | -0.13  (-0.26, 0.007) | 0.06 |  |
| Aetiology^≈^ | -0.18  (-4.12, 3.77) | 0.93 | -2.58  (-8.39, 3.22) | 0.38 | -0.11  (-1.11, 0.89) | 0.83 | 0.12  (-0.95, 1.19) | 0.83 |  |
| Contralateral joint replacement^❖^ | 1.34  (-0.83, 3.50) | 0.23 | 0.92  (-2.27, 4.11) | 0.57 | -0.50  (-2.06, 1.06) | 0.53 | -0.96  (-2.64, 0.72) | 0.26 |  |
| Prosthesis type^§^ | 1.04  (-0.96, 3.04) | 0.31 | 2.10  (-0.83, 5.04) | 0.16 |  |  |  |  |  |
| Patella  resurfaced | 1.37  (-1.00, 3.73) | 0.26 | 1.70  (-1.78, 5.18) | 0.34 |  |  |  |  |  |
| Cemented  prosthesis^$^ |  |  |  |  | -0.34  (-1.98, 1.31) | 0.69 | **2.38**  (0.61, 4.16) | 0.008 |  |
| Discharged home | 0.80  (-1.94, 3.54) | 0.60 | **5.82**  (1.83, 9.81) | 0.004 | **2.59**  (0.82, 4.36) | 0.004 | **4.78**  (2.89, 6.67) | <0.0001 |  |
| Complication | **-5.02**  (-7.49, -2.55) | <0.0001 | -2.33  (-5.96, 1.30) | 0.21 | -0.15  (-1.70, 1.40) | 0.85 | -0.09  (-1.75, 1.58) | 0.92 |  |
| Non-English speaking | **-4.76**  (-7.65, -1.87) | 0.001 | -3.53  (-7.77, 0.71) | 0.10 | -0.75  (-3.00,1.50) | 0.51 | 0.10  (-2.31, 2.51) | 0.93 |  |
| Number of Co-morbidities | -0.35  (-1.14, 0.43) | 0.37 | 0.22  (-0.93, 1.38) | 0.71 | -0.49  (-0.99, 0.01) | 0.06 | **-0.55**  (-1.09, -0.01) | 0.046 |  |
| ASA score^✪^ | 0.82  (-1.20, 2.85) | 0.42 | 0.81  (-2.17, 3.78) | 0.60 | -0.04  (-1.36, 1.28) | 0.95 | 0.25  (-1.17, 1.67) | 0.73 |  |
| Age-adjusted CCI* | **-0.76**  (-1.31, -0.20) | 0.008 | -0.77  (-1.58, 0.05) | 0.064 | -0.04  (-0.43, 0.35) | 0.85 | **-0.43**  (-0.86, -0.01) | 0.043 |  |
| Pre-op KSS_Pain_^∞^ | - | - | **-0.46**  (-0.65, -0.27) | <0.0001 |  |  |  |  |  |
| Pre-op KSS_Function_^**^ | **-0.10**  (-0.16, -0.04) | 0.001 | **-** | - |  |  |  |  |  |
| Pre-op HHS_Pain_^#^ |  |  |  |  | - | - | **-0.44**  (-0.61, -0.28) | <0.0001 |  |
| Pre-op HHS_Function_^¢^ |  |  |  |  | **-0.14**  (-0.23, -0.06) | 0.001 | - | - |  |
| Pre-op SF12_Physical_ | -0.09  (-0.27, 0.08) | 0.30 | 0.05  (-0.21, 0.31) | 0.72 | 0.07  (-0.07, 0.20) | 0.33 | -0.10  (-0.25, 0.04) | 0.15 |  |
| Pre-op SF12_Mental_ | **0.12**  (0.02, 0.21) | 0.017 | -0.09  (-0.22, 0.05) | 0.21 | **0.08**  (0.02, 0.14) | 0.015 | **-0.07**  (-0.14, -0.01) | 0.026 |  |

## ^⌘^SEIFA – Socioeconomic Index for Area, (0 to 10) with a higher score representing socioeconomic advantage

## ^¶^ BMI - Body Mass Index (weight [Kg] / height [m]^2^)

^≈^ coefficient relates to OA (compared with RA or AVN or CHD); OA – Osteoarthritis, RA – Rheumatoid Arthritis, AVN – Avascular necrosis, CHD – Congenital hip dysplasia

^❖^ contralateral knee joint replacement for the knee dataset and contralateral hip joint replacement for the hip dataset

^§^ coefficient relates to cruciate retaining (compared with posterior stabilizing or ultra-congruent) procedure

^$^ coefficient relates to cemented (hybrid or totally cemented) compared with uncemented hip replacement

^✪^ASA Score – American Anesthesiologist Society (1 to 4), with a higher score indicating greater systemic disturbance/operative risk

*CCI – Charlson Comorbidity Index (0-43, age adjusted), with a higher score indicating a greater comorbidity burden

^∞^KSS_Pain_ – Knee Society Pain Score (0 to 50) with a higher score representing less pain

^**^KSS_Function_ – Knee Society Function Score (0 to 100) with a higher score representing better function

^#^HHS_Pain_ – Harris Hip Pain Score (0 to 44) with a higher score representing less pain

^¢^HHS_Function_ – Harris Hip Function Score (0 to 47) with a higher score representing better function

## Table b) Predictors of SF12_Physical_ and SF12_Mental_ for the knee and hip datasets (multiple linear regression models) using change in scores as the dependent variable

|  | **Knee SF12_Physical_** | | **Knee SF12_Mental_** | | **Hip SF12_Physical_** | | **Hip SF12_Mental_** | |
| --- | --- | --- | --- | --- | --- | --- | --- | --- |
| **Variables** | coefficient  (95% CI) | *p* | coefficient  (95% CI) | *p* | coefficient  (95% CI) | *p* | coefficient  (95% CI) | *p* |
| Female sex | -0.66  (-2.09, 0.77) | 0.37 | 0.53  (-1.16, 2.22) | 0.54 | 1.29  (-0.20, 2.78) | 0.09 | -0.53  (-2.28, 1.22) | 0.56 |
| Age | -0.07  (-0.16, 0.01) | 0.08 | 0.012  (-0.08, 0.11) | 0.80 | -0.06  (-0.17, 0.04) | 0.23 | -0.03  (-0.15, 0.09) | 0.58 |
| SEIFA^⌘^ | 0.18  (-0.06, 0.42) | 0.15 | **-0.36**  (-0.65, -0.08) | 0.013 | 0.25  (-0.01, 0.51) | 0.06 | -0.15  (-0.46, 0.15) | 0.32 |
| BMI^¶^ | -0.06  (-0.17, 0.06) | 0.32 | 0.016  (-0.12, 0.15) | 0.82 | -0.04  (-0.17, 0.10) | 0.57 | 0.12  (-0.03, 0.28) | 0.12 |
| Aetiology^≈^ | -0.01  (-2.50, 2.48) | 0.99 | -0.44  (-3.39, 2.50) | 0.77 | -0.35  (-1.43, 0.72) | 0.52 | 0.62  (-0.64, 1.88) | 0.33 |
| Contralateral joint replacement^❖^ | 0.46  (-0.90, 1.83) | 0.51 | -1.09  (-2.70, 0.52) | 0.19 | -2.00  (-3.69, -0.32) | 0.02 | -0.32  (-2.29, 1.65) | 0.75 |
| Prosthesis type^§^ | 1.24  (-0.02, 2.50) | 0.054 | 1.13  (-0.36, 2.62) | 0.14 |  |  |  |  |
| Patella resurfaced | -0.06  (-1.56, 1.43) | 0.93 | 0.73  (-1.03, 2.50) | 0.42 |  |  |  |  |
| Cemented  prosthesis^$^ |  |  |  |  | 0.33  (-1.45, 2.11) | 0.72 | 0.36  (-1.72, 2.44) | 0.74 |
| Discharged home | **2.27**  (0.54, 4.01) | 0.01 | 0.14  (-1.90, 2.18) | 0.90 | **4.19**  (2.29, 6.10) | <0.0001 | 1.70  (-0.53, 3.94) | 0.13 |
| Complication | **-2.32**  (-3.89, -0.76) | 0.004 | **-1.89**  (-3.73, -0.05) | 0.044 | -0.96  (-2.63, 0.70) | 0.26 | -0.60  (-2.55, 1.36) | 0.55 |
| Non-English speaking | -1.31  (-3.14, 0.52) | 0.16 | 0.01  (-2.14, 2.16) | 0.99 | -0.31  (-2.73, 2.11) | 0.80 | 1.71  (-1.11, 4.53) | 0.23 |
| Number of Co-morbidities | -0.15  (-0.65, 0.34) | 0.55 | **-0.83**  (-1.41, -0.24) | 0.006 | **-0.62**  (-1.15, -0.08) | 0.025 | 0.35  (-0.28, 0.97) | 0.28 |
| ASA score^✪^ | 0.09  (-1.18, 1.37) | 0.89 | -0.10  (-1.61, 1.42) | 0.90 | -0.72  (-2.15, 0.70) | 0.32 | -0.83  (-2.50, 0.84) | 0.33 |
| Age-adjusted CCI^*^ | **-0.47**  (-0.82, -0.12) | 0.01 | -0.02  (-1.61, 1.42) | 0.92 | -0.37  (-0.79, 0.05) | 0.09 | -0.36  (-0.85, 0.14) | 0.16 |
| Pre-op KSS_Pain_^∞^ | **-0.15**  (-0.24, -0.07) | <0.0001 | -0.06  (-0.15, 0.04) | 0.27 |  |  |  |  |
| Pre-op KSS_Function_^**^ | 0.01  (-0.03, 0.05) | 0.50 | **-0.13**  (-0.18, -0.09) | <0.0001 |  |  |  |  |
| Pre-op HHS_Pain_^#^ |  |  |  |  | **-0.26**  (-0.43, -0.09) | 0.002 | **-0.21**  (-0.41, -0.01) | <0.041 |
| Pre-op HHS_Function_^¢^ |  |  |  |  | -0.02  (-0.12, 0.07) | 0.61 | **-0.39**  (-0.50, -0.29) | <0.0001 |
| Pre-op SF12_Physical_ | - | - | **0.43**  (0.31, 0.56) | <0.0001 | - | - | **0.64**  (0.47, 0.80) | <0.0001 |
| Pre-op SF12_Mental_ | **0.25**  (0.19, 0.30) | <0.0001 | - | - | **0.29**  (0.22, 0.35) | <0.0001 | - | - |

## ^⌘^SEIFA – Socioeconomic Index for Area, (0 to 10) with a higher score representing socioeconomic advantage

## ^¶^ BMI - Body Mass Index (weight [Kg] / height [m]^2^)

^≈^ coefficient relates to OA (compared with RA or AVN or CHD); OA – Osteoarthritis, RA – Rheumatoid Arthritis, AVN – Avascular necrosis, CHD – Congenital hip dysplasia

^❖^ contralateral knee joint replacement for the knee dataset and contralateral hip joint replacement for the hip dataset

^§^ coefficient relates to cruciate retaining (compared with posterior stabilizing or ultra-congruent) procedure

^$^ coefficient relates to cemented (hybrid or totally cemented) compared with uncemented hip replacement

^✪^ASA Score – American Anesthesiologist Society (1 to 4), with a higher score indicating a greater comorbidity burden

^*^CCI – Charlson Comorbidity Index (0-43, age adjusted), with a higher score indicating a greater comorbidity burden

^∞^KSS_Pain_ – Knee Society Pain Score (0 to 50) with a higher score representing less pain

^**^KSS_Function_ – Knee Society Function Score (0 to 100) with a higher score representing better function

^#^HHS_Pain_ – Harris Hip Pain Score (0 to 44) with a higher score representing less pain

^¢^HHS_Function_ – Harris Hip Function Score (0 to 47) with a higher score representing better function
